# Two-center validation of Pilot Tone Based Cardiac Triggering of a Comprehensive Cardiovascular Magnetic Resonance Examination

**DOI:** 10.21203/rs.3.rs-3121723/v1

**Published:** 2023-07-03

**Authors:** Yue Pan, Juliet Varghese, Matthew S. Tong, Vedat O. Yildiz, Alessia Azzu, Peter Gatehouse, Rick Wage, Sonia Nielles-Vallespin, Dudley Pennell, Ning Jin, Mario Bacher, Carmel Hayes, Peter Speier, Orlando P. Simonetti

**Affiliations:** The Ohio State University; The Ohio State University; The Ohio State University Wexner Medical Center; The Ohio State University; Royal Brompton Hospital; Royal Brompton Hospital; Royal Brompton Hospital; Royal Brompton Hospital; Royal Brompton Hospital; Siemens Medical Solutions USA; Siemens Healthcare GmbH; Siemens Healthcare GmbH; Siemens Healthcare GmbH; The Ohio State University

**Keywords:** cardiovascular MRI, Pilot Tone, cardiac triggering, prospective triggering

## Abstract

**Background:**

The electrocardiogram (ECG) signal is prone to distortions from gradient and radiofrequency interference and the magnetohydrodynamic effect during cardiovascular magnetic resonance imaging (CMR). Although Pilot Tone Cardiac (PTC) triggering has the potential to overcome these limitations, effectiveness across various CMR techniques has yet to be established.

**Purpose:**

To evaluate the performance of PTC triggering in a comprehensive CMR exam.

**Methods:**

Fifteen volunteers and twenty patients were recruited at two centers. ECG triggered images were collected for comparison in a subset of sequences. The PTC trigger accuracy was evaluated against ECG in cine acquisitions. Two experienced readers scored image quality in PTC-triggered cine, late gadolinium enhancement (LGE), and T1- and T2-weighted dark-blood turbo spin echo (DB-TSE) images. Quantitative cardiac function, flow, and parametric mapping values obtained using PTC and ECG triggered sequences were compared.

**Results:**

Breath-held segmented cine used for trigger timing analysis was collected in 15 volunteers and 14 patients. PTC calibration failed in three volunteers and one patient; ECG trigger recording failed in one patient. Out of 1987 total heartbeats, three mismatched trigger PTC-ECG pairs were found. Image quality scores showed no significant difference between PTC and ECG triggering. There was no significant difference found in quantitative measurements in volunteers. In patients, the only significant difference was found in post-contrast T1 (p = 0.04). ICC showed moderate to excellent agreement in all measurements.

**Conclusion:**

PTC performance was equivalent to ECG in terms of triggering consistency, image quality, and quantitative image measurements across multiple CMR applications.

## Introduction

Synchronization of cardiovascular magnetic resonance (CMR) data acquisition to cardiac motion is typically required to obtain artifact-free images of the heart and flowing blood. Conventionally, the R-wave of the electrocardiogram (ECG) is used as a trigger signal to synchronize data acquisition with the cardiac cycle. However, ECG setup can be time consuming; skin preparation is required and electrodes may need to be repositioned several times to obtain an adequate signal, thereby adding to the overall exam time [1]. The ECG signal also is prone to distortion caused by gradient and radiofrequency (RF) interference as well as the magnetohydrodynamic effect [2–4]. When the ECG fails, triggering based on peripheral pulse oximetry is often used as a backup; however, this method is susceptible to finger movement [5], frequently fails in patients with poor peripheral circulation, and an unpredictable trigger delay makes it difficult to adjust pulse sequence timing [6]. Cardiac triggering using other external biosensors have been proposed to overcome the limitations of ECG synchronization [6–11]. However, these methods typically require additional investigative hardware and operator training, making them less practical in a non-research setting. Self-gating (SG) cardiac synchronization extracts a cardiac motion signal from central k-space lines acquired at a constant time interval, eliminating the need for external gating hardware [12–14]. However, SG res continuous acquisition and consistent contrast, thus its applications are limited primarily to 3D and dynamic imaging. Changes in RF and gradient pulses can also distort the SG signal [15], and the need to acquire additional central k-lines can prolong acquisition time [16].

The Pilot Tone (PT) technique was proposed as a novel method to synchronize CMR acquisition with cardiac and respiratory motion [17]. The PT is a continuous RF signal generated by a small loop antenna positioned close to or inside the scanner bore, or mounted to a receiver coil. The frequency of the PT signal is within the bandwidth of the MR receiver system, but outside of the imaging bandwidth [17–19]. Hence, the PT does not disrupt the timing of the CMR pulse sequence, nor does it induce image artifacts. The PT is modulated by physiological motion and can be detected by the MR receiver coils to extract both respiratory and cardiac motion signals [20]. The cardiac component is highly correlated with cardiac contractile motion and can be viewed as a surrogate of ventricular volume [18] and used as a contact-free alternative to the ECG to synchronize data acquisition. The feasibility of retrospective and prospective PT cardiac (PTC) triggering has been previously demonstrated [21], and a fully automatic real-time PTC research framework was subsequently integrated with a variety of CMR applications. The objective of this exploratory study is to evaluate the feasibility of performing a comprehensive CMR exam with PTC triggering, and compare its performance with standard ECG synchronization in healthy volunteers and in patients referred for clinical imaging.

## Materials and Methods

### Study population

Fifteen healthy volunteers (36.8 ± 14.7 years, 7 females) and 20 cardiac patients (44.0 ± 15.9 years, 8 females) were recruited from two CMR centers (15 volunteers and 6 patients from Site A; 14 patients from Site B). The study was approved by the respective local institutional review boards and informed written consent was obtained from all subjects. The exclusion criteria for volunteers included: 1) under 18 years of age, 2) pregnant, 3) claustrophobic, and 4) standard contraindications to CMR. All cardiac patients were referred for CMR by local physicians. Demographic information for volunteers and patients is included in Supplementary Material 1.

### PTC signal extraction and trigger detection

Raw PT signals were acquired using the imaging receiver coils, simultaneous with image acquisition, and sampled at a rate of 2000 Hz. Detuning of the receiver coils during RF transmission, as is normally performed to avoid damage to the receiver system, induced a spike-like artifact in the PT signal. To eliminate this interference and to extract cardiac motion, a previously described PTC signal-processing algorithm [22] was employed as follows. A calibration scan consisting of four RF pulse trains (SINC shaped envelope, 1 ms duration, 70° flip angle, 40 pulses, 4ms between pulses) was performed, with a two second gap between pulse trains. RF interference was characterized by averaging and debiasing the PT samples over the RF pulse train repetitions. Principal component analysis (PCA) was applied, and the first two eigenvectors were stored as RF artifact subspace. Next, raw PT signals from a 20s free breathing undisturbed training scan were debiased and bandpass filtered with separate respiratory (0.2–0.6 Hz) and cardiac (0.8–2.5 Hz) frequency bands. PCA was applied to the respiratory filtered signal and the first two eigenvectors were stored as respiratory subspace. Finally, PCA was applied to the cardiac filtered signal and the first eigenvector orthogonal to both the RF artifact subspace and the respiratory subspace was calculated. The outcome of this approach yielded a channel combination vector that maximized the cardiac signal while suppressing respiratory contributions and RF artifacts. This vector was used in subsequent scans to combine unfiltered PT signals from all receiver coils into a single channel PT cardiac surrogate. A constant velocity Kalman filter was applied to the PT cardiac surrogate, and the first derivative taken to generate a denoised signal with no direct current offset and a latency of approximately 100 ms. This filtered first derivative signal enabled prospective triggering and is referred to as the PTC signal. The processing of the PTC did not assume regular rhythm; the only assumption made during the PTC calibration process was that the heartrate fell within the range of 48–150 beats per minute (0.8–2.5 Hz).

**Figure 1:**
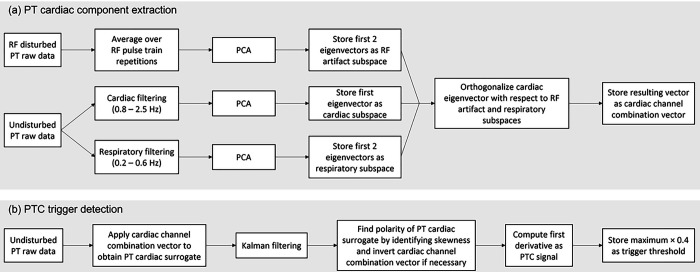
Diagram of PTC calibration including PT cardiac surrogate extraction (a) and PTC trigger detection (b). For PT cardiac surrogate extraction (a), raw PT calibration data without RF interference is band pass filtered to enhance cardiac and respiratory signals, and raw PT calibration data with RF interference is averaged to enhance RF pulse artifacts to stabilize identification of the corresponding subspaces by PCA. Subspaces of unwanted signal contributions for RF artifacts and respiration are identified by their first two eigenvectors. The cardiac subspace is identified by its first eigenvector and unwanted contributions to the combined signal are minimized by orthogonalizing the vector with respect to both subspaces, resulting in the final cardiac channel combination vector. For trigger detection (b) the raw PT calibration data without RF interference is processed the same way as the continuous raw PT data stream during triggered measurements: raw PT data is combined using the cardiac channel combination vector to form the PT cardiac surrogate, then a constant velocity Kalman filter and the first derivative are applied to generate a PTC signal with low latency which served as the output waveform seen on the scanner user interface and on which the trigger is detected. During calibration only, the polarity of the PT cardiac surrogate is determined by identifying the skewness of the Kalman outputs, since diastole has a longer duration than systole. The average over the local maxima of the PTC signal is stored for signal scaling, and the triggering threshold was set to be 0.4 times of the stored value.

The polarity of the PT cardiac surrogate was determined during calibration based on skewness. Under resting conditions, diastole is longer than systole, thus the skewness indicates the direction of diastole and can be used to define the polarity of the PTC. The maximum of the PTC signal acquired during the calibration scan was also stored, and the threshold for triggering set at 0.4 times this maximum. This threshold was set empirically as a compromise between reliably avoiding double triggers caused by the mid-diastolic “shoulder” of the signal, while simultaneously avoiding missing triggers due to low signal maxima. A diagram of the PTC calibration process is summarized in Fig. 1.

The delay between the PTC trigger and ECG R-wave can be estimated during calibration by assuming that the PT cardiac surrogate reaches its maximum simultaneous with the R-wave. The delay was calculated as the average of the times between the PT trigger points in the training data and their preceding PT cardiac surrogate local maxima, plus the processing latency induced by Kalman filter. This delay estimation was later used to automatically set the acquisition window for single-shot scans, which are typically timed to diastole.

### Image acquisition

Data were collected on two clinical 3T systems (MAGNETOM Vida, software version XA31, Siemens Healthcare, Erlangen, Germany) at two different centers. A 20-second PT calibration scan was performed on each subject before acquiring PTC triggered images. All breath-held scans were acquired at end expiration.

At Site A, 15 healthy volunteers underwent a comprehensive, PTC triggered, non-contrast cardiac exam that included acquisition of cardiac localizer, dark-blood turbo spin echo (DB-TSE), cine covering the left and right ventricles, flow of the ascending aorta and the main pulmonary artery (MPA), and quantitative mapping images. Corresponding ECG triggered images for each application were also collected for comparison in these volunteers. Additionally, in six patients clinically referred for CMR at Site A, PTC triggered pre- and post-contrast T1 maps and late gadolinium enhancement (LGE) images were acquired along with corresponding ECG triggered scans. Scan parameters and slice positions were matched between PTC and ECG triggered sequences, although the repetition times (TR) of the PTC triggered scans were rounded up to the nearest 0.5 ms to match the PT sampling rate. Inversion time in PTC triggered LGE scans was set slightly longer relative to the ECG triggered scans to account for the longer time delay between contrast injection and image acquisition. A summary of sequences and scan parameters used at Site A are shown in Supplementary Material 2.

At Site B, 14 cardiac patients clinically referred for CMR were scanned. All CMR sequences were PTC triggered, except for an additional set of ECG-triggered cine slices collected for comparison. The specific CMR sequences used on each patient varied depending on the clinical indication; these included sequences for localization, morphology, cine, flow, parametric mapping, early gadolinium enhancement (EGE), and LGE. A summary of sequences and scan parameters used at Site B are shown in Supplementary Material 3.

### Trigger analysis

PTC and ECG triggered, breath-hold segmented cine series were acquired in the majority of subjects, including 15 of the volunteers at Site A, and 14 of the patients at Site B. These data were used to evaluate PTC trigger accuracy. During each PTC triggered image acquisition, the ECG signal and triggers were recorded simultaneously by the scanner. PTC and ECG trigger timing were evaluated using MATLAB (MathWorks, Natick, MA, USA). The first heartbeat was not used to acquire image data; therefore, the first trigger was excluded from the analysis. PTC and its corresponding ECG triggers were identified. The R-wave based ECG trigger was assumed to always occur prior to the motion-based PTC trigger, as electrical activity of the heart precedes mechanical motion. Therefore, trigger delay was defined as the time elapsed from the ECG trigger to the PTC trigger within each heartbeat. Trigger jitter was defined as the standard deviation of the trigger delays across heartbeats. Both mean trigger delay and trigger jitter were calculated in milliseconds and as a fraction of average cardiac cycle measured by ECG (% RR_ECG_).

### Image qualitative analysis

To qualitatively evaluate the image sharpness and artifact level, matching cine, LGE, T1- and T2-weighted DB-TSE images acquired with both triggering methods were scored by two experienced CMR readers (30 and 8 years of experience); these images came from subsets of volunteers and patients at each center, depending on the availability of PTC and ECG comparative data. Images from all cardiac views from a single subject, single application, and single triggering method were combined into a single display and presented in random order to the readers who were blinded to the triggering method. The scoring criteria ranging from 5: excellent, no apparent artifacts and/or blurring to 1: very poor, image totally obstructed by artifacts and/or blurring. In 6 patients from Site A with both ECG and PTC triggered LGE, the images were randomized and the global presence/absence of LGE was evaluated by a physician.

### Image quantitative analysis

Cardiac function and flow measurements were quantitatively evaluated using suiteHEART (NeoSoft, Pewaukee, WI, USA). Biventricular cardiac output (CO), stroke volume (SV), ejection fraction (EF), and end systolic and end diastolic volumes (ESV, EDV) were measured from cine images acquired in both volunteers (Site A) and patients (Site B). Aortic and MPA CO, SV, and peak velocities were measured from phase contrast images. Myocardial T1, T2, and T2* relaxation times were measured in the interventricular septum from parametric maps in volunteers, per SCMR guidelines [23]. In patients scanned at Site A, where both PTC and ECG triggering was performed, pre and post contrast T1 values were measured in the interventricular septum, and extracellular volume (ECV) fractions were calculated.

### Statistical analysis

The Wilcoxon signed rank test with an alpha level of 0.05 was performed on the image quality scores to test for differences between the two triggering methods. Pairwise Student’s t-test with an alpha level of 0.05 was performed on the quantitative measurements to test for significant differences. The p-values of biventricular cardiac function measurements, aortic flow, MPA flow, and parametric mappings were adjusted separately using the Benjamini and Hochberg correction method [24] with a false discovery rate of 0.05. Two-way mixed effects, absolute agreement, single rater intra-class correlation coefficients (ICC) were also computed to test the agreement between the two triggering methods. An ICC of 0.9–1 indicated excellent agreement; 0.75–0.89 indicated good agreement; 0.5–0.74 indicated moderate agreement; and 0–0.49 indicated poor agreement.

## Results

**Figure 2:**
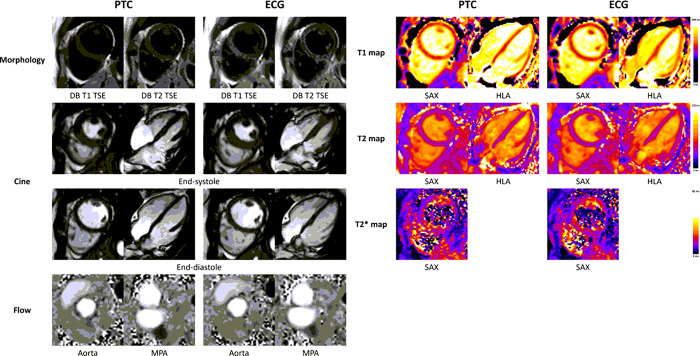
Comparison between PTC and ECG triggered images in a healthy volunteer PTC triggered images were acquired including morphology, segmented cine covering the left and right ventricles, segmented flow of the ascending aorta and the main pulmonary artery (MPA), quantitative myocardial relaxation time maps. Subject has a heartrate around 90 beats per minute. Corresponding ECG triggered images were also collected using identical scan parameters and slice positions for comparison.

**Figure 3:**
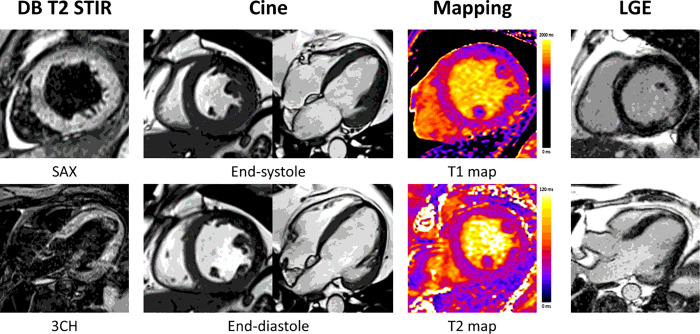
An example of PTC triggered images of a cardiac patient. PTC triggered images were acquired including morphology, segmented cine covering the left and right ventricles, quantitative myocardial relaxation time maps, and LGE images. Subject was referred to an CMR exam for myocardial sarcoidosis. Subject was severely obese with EF less than 35%, and has a heartrate around 80 beats per minute.

Figure 2 shows images for each CMR sequence acquired using both triggering methods in a healthy volunteer scanned at Site A, and Fig. 3 shows PTC triggered images in a patient scanned at Site B. Movies of cine and flow image series in the healthy volunteer can be found in Supplementary Material 4 and 5. The number of subjects included in each analysis is listed in Table 1. Volunteer and patient data were analyzed separately; as a result, data from the two centers were not combined in any of the subsequent analyses.

### Trigger analysis

**Figure 4:**
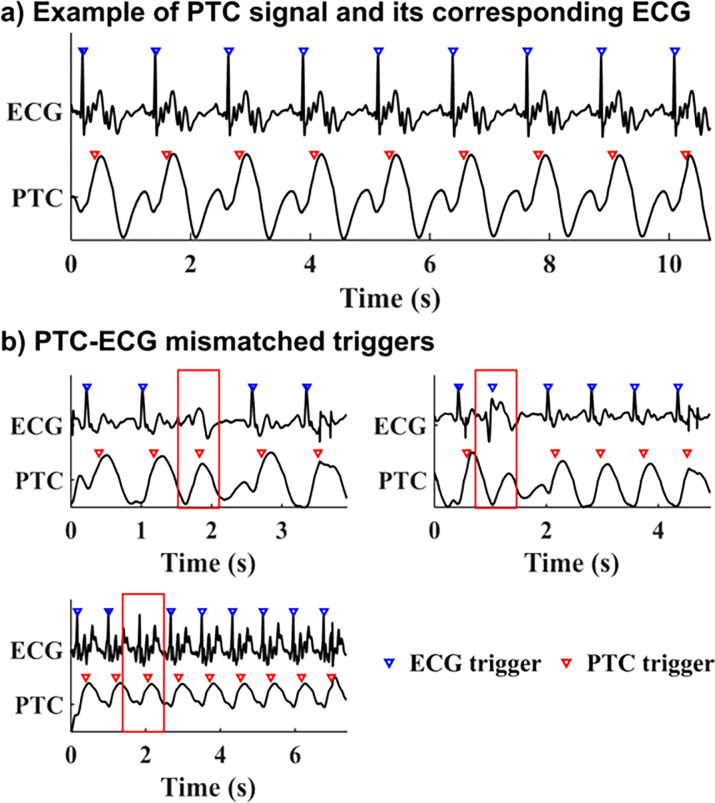
ECG vs. PTC signal recorded simultaneously during segmented cine acquisitions. Triangle represents the triggering of each heartbeat. A delay was observed in the PTC trigger with respect to ECG based R-wave triggering. This was expected as the R-wave precedes the mechanical contraction of the heart, upon which the PTC trigger was based. 4a) shows an example of successful PTC acquisition where no mismatching between ECG and PTC was found. 4b) shows all cases of mismatched PTC-ECG trigger pairs. Two out of three mismatching observed were due to arrhythmias in patient. Although ECG was used as reference standard, one mismatched trigger case was apparently due to ECG failure. Mismatched triggers are indicated by the red boxes.

**Figure 5:**
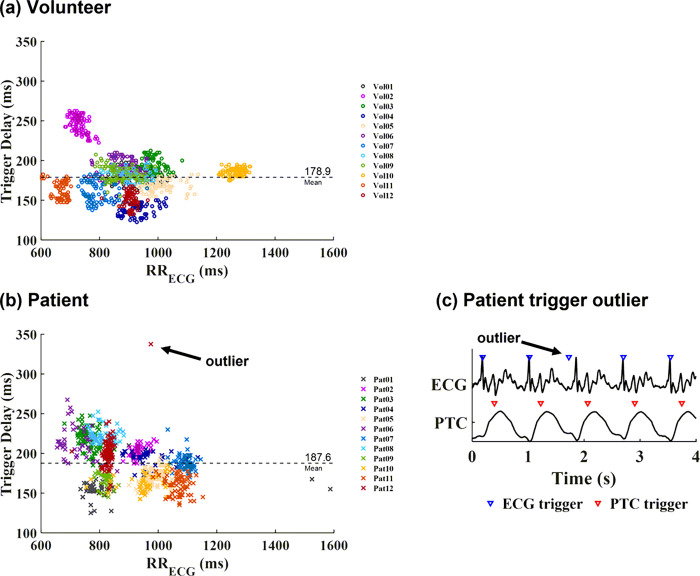
Trigger Delay (ms) vs. RR interval in (a) volunteers and (b) patients. The average trigger delay in volunteers was 178.9 ± 9.4 ms, 20.4 ± 1.5 %RR_ECG_. The average trigger delay in patients was 187.6 ± 12.5 ms, 21.5 ± 1.8 %RR_ECG_. The trigger delay was consistent regardless of the RR interval, especially within each individual. Trigger jitter was higher in patients. An abnormally long PTC trigger delay was pointed out as outlier in (b), which was due to incorrect early detection of ECG shown in (c).

An example of PTC vs. ECG signal and triggers are shown in Fig. 4a. PTC signal extraction failed in one volunteer at Site A who had dextrocardia with situs inversus. The recording of ECG trigger timing failed in one patient at Site B. The PTC signal was inverted in two volunteers and one patient, i.e., the skewness criteria to determine PT signal polarity failed, causing the trigger timing to be shifted to the time of diastolic filling. Although PTC triggered images were collected and scored for quality in these subjects, PTC triggering was considered to be a failure and these data were excluded from the trigger timing analysis. Out of 1987 PTC triggers from the remaining subjects (12 volunteers at Site A and 12 patients at Site B), three mismatches with ECG were found (Fig. 4b). The average trigger delay and trigger jitter in each subject are listed in Table 2. Figure 5 shows the trigger delay in milliseconds vs. RR interval for both healthy volunteers (5a) and patients (5b), as well as one example of an outlier (5c).

### Image qualitative analysis

A summary of the image quality analysis is shown in Table 3. No Images were acquired in one volunteer who had dextrocardia with situs inversus, causing PTC signal extraction to fail. Images from another volunteer were discarded due to gross motion observed between image series; poor correspondence of slice positions between the two triggering methods was observed, making comparison difficult. Cine images from the remaining 13 volunteers and 14 patients were randomized and scored by two readers; each image set included three long-axis views, and a stack of short-axis views covering the left and right ventricles. Wilcoxon signed rank test p-values for the two readers were 1 and 0.25 for volunteer data, and 1 and 0.63 for patient data, indicating no significant difference in cine image quality between triggering methods. LGE images from the six patients from Site A with both ECG and PTC triggering were scored in random order; each image set included three long-axis and three short-axis views. Wilcoxon signed rank test p-values were 1 for both readers, indicating no significant difference in LGE image quality scores between triggering methods. DB-TSE was not collected in any patients, and in five volunteers, ECG triggered DB-TSE was not acquired. T1- and T2-weighted DB-TSE images from the remaining eight volunteers were scored in random order; each image set included horizontal long axis and mid short axis views. Wilcoxon signed rank test p-values for T1-weighted DB-TSE were both 1, and p-values for T2-weighted DB-TSE were 0.68 and 1, again indicating no significant difference in DB-TSE image quality scores between triggering methods. LGE was present in three of the six patients and the findings from both triggering methods agreed.

### Image quantitative analysis

In volunteers, quantitative global cardiac function results for PTC and ECG triggered cine, flow from 2D phase contrast, and myocardial T1, T2, and T2* values from parametric maps are shown in Table 4. In five volunteers, ECG triggered MPA flow was not acquired, and in one volunteer ECG triggered parametric maps were not acquired. No significant difference was found between the two triggering methods in biventricular function, aortic and MPA flow, and parametric mapping values. The ICC demonstrated good to excellent concordance for most of the measurements, except for RV cardiac output, MPA peak velocity, and myocardial T2 and T2* values, which displayed moderate concordance.

Biventricular cardiac function values from cine, and native and post contrast T1 and ECV values in patients are shown in Table 5. No significant difference was found between the two triggering methods in cardiac function, native T1, and ECV; only post-contrast T1 showed a significant difference. ICC showed excellent agreement among all measurements.

## Discussion

This preliminary study assessed the feasibility of performing a comprehensive CMR exam with PTC triggering and compared its performance with ECG triggering. Although the PTC calibration process failed in some subjects, the PTC triggered acquisitions showed good agreement with ECG triggering in terms of consistency of trigger timing, image quality, and quantitative measurements. Complete CMR exams including a variety of applications were performed using PTC triggering in volunteers at Site A, and in patients at Site B. All patient studies were of diagnostic quality and the PTC triggered images were all read and reported without exception.

A delay was observed in the PTC trigger with respect to ECG based R-wave triggering. This was expected as the PTC trigger is based on the mechanical contraction of the heart, which is preceded by the R-wave. The length of delay varied somewhat between subjects, potentially due to subtle differences in cardiac anatomy and function; this variation was also observed in other PTC studies [20, 25]. The PTC processing induced an additional systematic delay which was consistent among subjects. This delay could vary from that reported in other studies due to the use of different processing algorithms (i.e., prospective vs. retrospective, filter length, and peak triggering vs. threshold triggering). Some beat-to-beat variation in PTC trigger delay, or “jitter”, measured relative to the ECG trigger, was observed. Taking the ECG trigger time as the gold standard, jitter was assumed to be purely caused by the instability of PTC. The worst-case jitter measured in any subject was less than the temporal resolution of the image acquisition. Jitter was worse in patients than in healthy volunteers. This may be due to alterations of excitation-contraction coupling [26], or dysfunction of electrical conduction pathways [27, 28] that can accompany cardiac disease. Since the PTC is dependent on the mechanical motion of the heart, which is ultimately what impacts CMR image quality, timing of data acquisition and image quality may actually be improved with PTC in cases where there is a mismatch between electrical activity and mechanical contraction [29].

Among the 24 subjects with successful PTC calibration, only three trigger discrepancies were found between PTC and ECG, and two of these appeared to be due to premature ventricular contraction (PVC). Arrhythmias can be problematic for cardiac synchronization regardless of the triggering technique [30]; however, given that the purpose of cardiac triggering is to synchronize data acquisition with cardiac motion, it is logical to assert that trigger detection based on a motion signal may be more effective than the electrical ECG signal. In a previous study [29], when both triggering methods were compared with cardiac motion extracted from real-time cine images, PTC was shown to have fewer mis-triggers than ECG, especially in patients with arrhythmias. Our designation of any discrepancy between PTC and ECG as an error in the PTC trigger was a conservative approach that did not consider this.

Although there was no difference in cine, LGE, and DB-TSE image quality scores between the two triggering methods in terms of blurring and artifacts, it is worth noting that the PTC triggered cine and flow series started at mid-systole, rather than end diastole as is the case for ECG triggering. This timing difference did not affect quantification of global cardiac function or flow, as standard commercial software can typically choose the end-systolic and end-diastolic frames [31], regardless of the timing of these frames within the image series.

In static imaging applications including parametric mapping, the trigger jitter was negligible compared to the duration of diastole and caused no significant difference in myocardial mapping values in volunteers. However, in patients the post contrast T1 values measured using PTC were found to be higher than those measured using ECG. Given that PTC images in this patient cohort were consistently obtained after conventional ECG images, the significance of this finding could largely be attributed to the difference in post-contrast injection timing.

The PTC delay time was estimated during calibration and the trigger timing automatically adjusted accordingly. However, this process may not be effective for the dark-blood preparation pulse which is typically applied at end-diastole (the ECG R-wave) with data acquisition timed to late diastole of the same cardiac cycle [32]; a mismatch in this timing could lead to myocardial signal loss. While the trigger timing of DB-TSE was set automatically in this manner, minimal cardiac motion artifact was observed. The majority of artifacts were related to respiratory motion, and these were seen in both PTC and ECG triggered images.

The PTC requires a small loop antenna known as the PT generator; such a device has been embedded in the anterior body array coil of several current Siemens MRI systems operating at various field strengths. No hardware or software modifications are required to obtain PTC signals on these scanners. No additional patient preparation is required beyond positioning the coil so that the PT generator is placed directly above the heart. At the time of the study, PTC signal and triggering were detected using a research algorithm, which is now commercially available as Beat Sensor^®^. The elimination of the time associated with ECG lead placement and the challenges of ECG interference, especially at higher field, make the PTC a promising cardiac synchronization technique.

While the potential advantages of PTC are substantial, this study has several limitations. The small sample size limits the strength of conclusions that can be drawn from a number of comparisons. LGE and parametric maps with both triggering methods were available in only six patients from one center; no definite conclusions should be drawn based on such a limited sample. It must also be noted that PTC calibration failed in several subjects. PTC signal polarity was inverted in three subjects with high heart rates, causing the PTC trigger time to be shifted to peak diastolic filling rather than peak systolic contraction, introducing additional delay relative to the R-wave. This difference in trigger timing did not impact cine, flow, and parametric mapping image analyses, since the delay was consistent from beat-to-beat. However, a more robust determination of PT signal polarity is desirable. Poor PTC performance in the subject with situs inversus may have been due to the position and orientation of the heart relative to the PT generator, and perhaps could have been corrected by repositioning the coil. Although the PTC algorithm has been improved since the time of this study, adjusting the coil position and recalibrating may still be required. If these steps do not improve PTC triggering, then falling back to conventional triggering methods should be considered.

The current study included three patients with BMI over 40, and two patients with EF less than 35%, including one patient in whom both factors coexisted. No issues were observed in PTC triggered sequences in these patients. Although the patients in this study were referred for CMR with a variety of indications, further evaluation is needed in patients with a range of body habitus, cardiac orientations, and heart rates, and in various conditions that can alter cardiac motion and blood flow. The motion-derived PT signal could be attenuated in severely obese or small pediatric patients, or in patients with heart failure with reduced EF, or ventricular dyssynchrony. On the other hand, ECG triggering may be challenging in patients with conduction system disorders. Further investigation is required to compare the performance of PTC to ECG under the above circumstances.

## Conclusion

PTC triggering was successfully evaluated across a wide range of CMR applications in healthy volunteers and in cardiac patients at two unaffiliated imaging centers. PTC performance was compared with standard ECG triggering and found to provide accurate triggering as well as comparable image quality and quantitative results. PTC may offer a more efficient and effective method than ECG for CMR cardiac synchronization.

## Figures and Tables

**Table 1:** **List of the number of subjects included for each analysis.** For the trigger analysis, PTC calibration failed in three volunteers and one patient. ECG trigger recording also failed in one patient. For image analyses, two volunteers were excluded due to unsuccessful image collection and gross motion observed between image series. In one volunteer, ECG-triggered parameter mapping was not acquired. Additionally, in five volunteers, ECG-triggered DB-TSE and MPA flow were not collected. Although PTC triggering was used for the entire CMR exam in all patients at Site B, corresponding ECG triggering was only acquired for cine images.

Number of Subjects Included		Volunteer	Patient	
		Site A	Site A	Site B
**Total**		15	6	14
**Trigger Analysis**		12	-	12
**Image Qualitative/Quantitative Analysis**	**DB-TSE**	8	-	-
	**Cine**	13	-	14
	**Aortic Flow**	13	-	-
	**MPA Flow**	8	-	-
	**Mapping**	12	6	-
	**LGE**	-	6	-

**Table 2:** **List of triggering analysis results for each individual.** PTC triggered segmented cine data was used for the analysis; all slices were included. ECG signals and triggers were acquired simultaneously. Trigger delay was defined as the time elapsed from the ECG trigger to the PTC trigger, and were calculated using matched trigger pairs only. Trigger jitter was the standard deviation of the trigger delays across heartbeats

Volunteer	# Matched Triggers	# Mismatched Triggers	RR_ECG_ (ms)	Trigger Delay ± Trigger Jitter (ms)	Trigger Delay ± Trigger Jitter (% RR_ECG_)
Vol01	94	0	919.4	181.3 ±10.2	19.7 ± 1.7
Vol02	108	0	732.0	246.0 ±10.8	33.6 ± 2.2
Vol03	91	0	984.5	194.5± 9.5	19.7 ± 1.2
Vol04	84	0	928.9	138.5± 8.0	14.9 ± 1.0
Vol05	98	0	1003.0	167.3 ±9.1	16.8 ± 1.0
Vol06	104	0	873.3	192.8 ± 10.1	22.1 ± 1.6
Vol07	98	0	794.3	159.7 ±12.4	20.1 ± 2.0
Vol08	87	0	903.8	183.9 ±6.8	20.4 ± 1.4
Vol09	90	0	868.6	183.1 ±9.8	21.1 ± 1.9
Vol10	94	0	1265.7	186.4 ±5.0	14.7 ± 0.4
Vol11	84	0	668.1	162.4 ±10.8	24.4 ± 2.0
Vol12	83	0	905.1	150.7 ±9.8	16.7 ± 1.1
**Total**	**1115**	**0**	-	-	-
**Average**	-	-	**903.9**	**178.9 ±9.4**	**20.4 ± 1.5**
Patient	# Matched Triggers	# Mismatched Triggers	RR_ECG_ (ms)	Trigger Delay (ms)	Trigger Delay (% RR_ECG_)
Pat01	60	2	819.5	154.4 ± 12.4	19.3 ± 2.6
Pat02	58	0	932.3	202.8 ± 7.3	21.8 ± 0.7
Pat03	95	0	765.5	217.6± 14.2	28.5 ± 2.6
Pat04	59	0	970.2	191.4 ± 10.7	19.8 ± 1.7
Pat05	107	0	987.3	169.4 ± 11.2	17.2 ± 1.1
Pat06	55	0	724.9	214.8 ± 22.5	29.6 ± 3.8
Pat07	79	0	1091.9	187.6 ± 11.4	17.1 ± 1.1
Pat08	68	0	811.7	223.2 ± 9.7	27.6 ± 1.7
Pat09	52	0	820.2	165.7 ± 9.3	20.2 ± 1.3
Pat10	54	0	946.6	163.1 ± 10.6	17.2 ± 1.6
Pat11	81	0	1068.1	159.0 ± 11.3	14.9 ± 1.3
Pat12	101	1	829.7	202.5 ± 19.4	24.3 ± 2.0
**Total**	**869**	**3**	-	-	-
**Average**	-	-	**897.3**	**187.6 ± 12.5**	**21.5 ± 1.8**

**Table 3:** **Qualitative results scored by two experienced CMR readers.** Scores are reported as mean ± standard deviation. ECG triggered LGE images were only acquired in patients at Site A. DB-TSE was not collected in patients.

Image	Reader	PTC	ECG	p-value	PTC	ECG	p-value
		Volunteer (n = 13)			Patient (n = 14)		
**Cine**	1	4.9 ±0.3	4.8 ±0.6	1	4.4 ± 0.5	4.5 ± 0.8	1
	2	4.8 ±0.4	4.5 ±0.7	0.25	4.3 ± 0.7	4.4 ± 0.8	0.63
					**Patient (n = 6)**		
**LGE**	1				4.7 ± 0.5	4.5 ± 0.5	1
	2				4.7 ± 0.5	4.8 ± 0.4	1
		**Volunteer (n = 8)**					
**T1w DB-TSE**	1	4.1 ±0.6	4.3 ±0.5	1			
	2	4.5 ±0.5	4.8 ±0.5	1			
**T2w DB-TSE**	1	4.5 ±0.5	4.4 ± 1.1	0.68			
	2	4.6 ±0.5	4.8 ± 0.7	1			

**Table 4:** **Quantitative CMR results measured in volunteers.** Values are reported as mean ± standard deviation, and range (minimum - maximum). The pairwise Studenťs t-test showed no significant difference between the triggering methods. The ICC demonstrated good to excellent agreement between most measurements, except for right ventricular CO, MPA peak velocity, and T2 and T2* values, which showed only moderate agreement.

Volunteer		PTC	ECG	Adjusted P	ICC
**Segmented Cine (n = 13)**					
HR (bpm)		70 ± 12 (47–90)	69 ± 12 (47–92)	0.58	0.94
LV	CO (L/min)	5.6 ± 1.1 (3.7–7.7)	5.7 ± 1.2 (3.8–8.0)	0.97	0.90
	SV (mL)	82.3 ± 17.8 (56.6127)	83.7 ± 17.5 (54–126)	0.53	0.98
	EF (%)	56.8 ± 5.2 (50–67)	57.3 ± 5.7 (49–69)	0.56	0.94
	ESV (mL)	62.9 ± 15.1 (40.187.4)	62.8 ± 14.9 (39.486.3)	0.97	0.98
	EDV (mL)	145.1 ± 29.3 (97.2200)	146.5 ± 28.2 (96.8199)	0.53	0.99
RV	CO (L/min)	5.4 ± 0.9 (4.2–7.2)	5.4 ± 1.0 (4.2–8)	0.97	0.72
	SV (mL)	78.4 ± 16.8 (46.7118)	80.0 ± 17.5 (53.4126)	0.53	0.98
	EF (%)	51.7 ± 5.8 (44–61)	51.7 ± 5.7 (44–62)	1	0.97
	ESV (mL)	75.9 ± 27.2 (38.7140)	77.1 ± 26.2 (43–136)	0.56	0.99
	EDV (mL)	154.4 ± 41.7 (85.4258)	157.2 ± 41.6 (96.4263)	0.53	0.99
**2D Flow**					
Aorta (n = 13)	HR (bpm)	69 ± 10 (61–94)	69 ± 11 (57–91)	0.80	0.87
	CO (L/min)	5.9 ± 0.9 (4.8–7.3)	5.9 ± 1.0 (4.7–7.4)	0.80	0.92
	SV (mL)	85.8 ± 15.7 (57.3114)	87.5 ± 18.3 (51.6120)	0.66	0.94
	Peak Velocity (cm/s)	122.8 ± 25.2 (94.9188)	128.3 ± 24.2 (99.9192)	0.14	0.92
MPA (n = 8)	HR (bpm)	70 ± 13 (58–93)	73 ± 11 (59–88)	0.36	0.78
	CO (L/min)	5.3 ± 0.8 (4.3–6.5)	5.5 ± 0.8 (4.8–6.7)	0.36	0.80
	SV (mL)	77.2 ± 14.6 (57.2101)	77.0 ± 15.1 (57.1101)	0.93	0.96
	Peak Velocity (cm/s)	81.5 ± 14.4 (65–99.6)	77.0 ± 14.1 (65.399.1)	0.36	0.72
**Mapping (n = 12)**				
T1 (ms)		1203 ± 43 (11121262)	1205 ± 35 (11481260)	0.74	0.87
T2 (ms)		38.1 ± 1.5 (36.2–41.5)	38.4 ± 2.0 (36.2–42.1)	0.67	0.74
T2* (ms)		23.1 ± 3.9 (18.3–30.4)	24.6 ± 6.1 (16.2–37.1)	0.67	0.54

Biventricular cardiac function values from cine, and native and post contrast T1 and ECV values in patients are shown in Table 5. No significant difference was found between the two triggering methods in cardiac function, native T1, and ECV; only post-contrast T1 showed a significant difference. ICC showed excellent agreement among all measurements.

**Table 5:** **Quantitative CMR results measured in patients.** Values are reported as mean ± standard deviation, and range (minimum - maximum). Only post contrast T1 values showed a significant difference between triggering methods. ICC showed excellent agreement among all measurements.

Patient		PTC	ECG	Adjusted P	ICC
**Segmented Cine (n = 14)**					
HR (bpm)		70 ± 10 (55–86)	71 ± 9 (57–86)	0.94	0.97
LV	CO (L/min)	6.4± 1.5 (3.5–8.8)	6.4 ± 1.6 (3.7–9.0)	0.94	0.99
	SV (mL)	92.4 ± 25.7 (47.6–135)	91.9 ± 25.6 (46.8–133)	0.94	0.99
	EF (%)	53.1 ± 13.6 (25–72)	52.7 ± 13.3 (25–72)	0.94	1.00
	ESV (mL)	80. ± 32.2 (26.1–141)	80.7 ± 30.8 (25.6–141)	0.94	0.99
	EDV (mL)	168.2 ± 43.0 (89.7–222)	168.1 ± 40.9 (92.6–217)	0.94	0.99
RV	CO (L/min)	5.0 ± 1.5 (3.3–7.8)	4.9 ± 1.4 (3.5–7.5)	0.94	0.98
	SV (mL)	72.0 ± 19.9 (48.7–114)	70.6 ± 19.4 (47.3–110)	0.94	0.99
	EF (%)	53.4 ± 9.7 (40–71)	53.2 ± 10.2 (40–70)	0.94	0.97
	ESV (mL)	62.4 ± 25.8 (25.7–110)	61.4 ± 25.1 (27.6–104)	0.94	0.98
	EDV (mL)	130.3 ± 38.6 (70.5–198)	129.7 ± 34.5 (79.0–185)	0.94	0.98
**Mapping (n = 6)**					
Native T1 (ms)		1223 ± 62 (1161–1325)	1225 ± 57 (1169–1329)	0.75	0.96
Post contrast T1 (ms)		549 ± 35 (513–590)	536 ± 41 (494–590)	**0.04**	0.93
ECV		25.7 ± 2.8 (22.3–30.0)	25.6 ± 2.9 (21.6–29.9)	0.75	0.99

## Abbreviations

BMI: body mass index
bSSFP: balanced steady state free precession
CMR: cardiovascular magnetic resonance
CO: cardiac output
DB-TSE: dark-blood turbo spin echo
ECG: electrocardiogram
EF: ejection fraction
EGE: early gadolinium enhancement
EDV/ESV: end diastolic/systolic volume
HR: heart rate
ICC: intra-class correlation coefficients
LGE: late gadolinium enhancement
MPA: main pulmonary artery
PCA: principal component analysis
PT: Pilot Tone
PTC: Pilot Tone Cardiac
RF: Radiofrequency
SG: self-gating
SV: stroke volume
TR: repetition time
